# Recoverability Analysis for Modified Compressive Sensing with Partially Known Support

**DOI:** 10.1371/journal.pone.0087985

**Published:** 2014-02-10

**Authors:** Jun Zhang, Yuanqing Li, Zhenghui Gu, Zhu Liang Yu

**Affiliations:** 1 College of Information Engineering, Guangdong University of Technology, Guangzhou, People's Republic of China; 2 Center for Brain Computer Interfaces and Brain Information Processing, South China University of Technology, Guangzhou, People's Republic of China; University of Bonn, Bonn-Aachen International Center for IT, Germany

## Abstract

The recently proposed modified-compressive sensing (modified-CS), which utilizes the partially known support as prior knowledge, significantly improves the performance of recovering sparse signals. However, modified-CS depends heavily on the reliability of the known support. An important problem, which must be studied further, is the recoverability of modified-CS when the known support contains a number of errors. In this letter, we analyze the recoverability of modified-CS in a stochastic framework. A sufficient and necessary condition is established for exact recovery of a sparse signal. Utilizing this condition, the recovery probability that reflects the recoverability of modified-CS can be computed explicitly for a sparse signal with 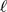 nonzero entries. Simulation experiments have been carried out to validate our theoretical results.

## Introduction

A central problem in CS is the following: given an 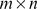 matrix 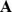 (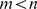), and a measurement vector 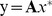, recover 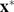. To deal with this problem, the most extensively studied recovery method is the 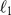-minimization approach (Basis Pursuit) [1]–[5]

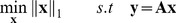
(1)This convex problem can be solved efficiently; moreover, 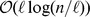 probabilistic measurements are sufficient for it to recover a 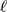-sparse vector 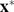 (i.e., all but at most 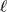 entries are zero) exactly.

Recently, Vaswani and Lu [6]–[9], Miosso [10], [11], Wang and Yin [12], [13], Friedlander et.al [14], Jacques [15] have shown that exact recovery based on fewer measurements than those needed for the 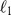-minimization approach is possible when the support of 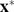 is partially known. The recovery is implemented by solving the optimization problem.
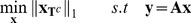
(2)where **T** denotes the “known” part of support, 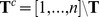, 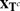 is a column vector composed of the entries of 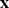 with their indices being in 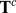. This method is named modified-CS [6] or truncated 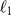 minimization [12]. One application of the modified-CS is the recovery of (time) sequences of sparse signals, such as dynamic magnetic resonance imaging (MRI) [8], [9]. Since the support evolve slowly over time, the previously recovered support can be used as known part for later reconstruction.

As an important performance index of modified-CS, its recoverability, i.e., when is the solution of (2) equal to 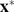, has been discussed in several papers. In [6], a sufficient condition on the recoverability was obtained based on restricted isometry property. From the view of *t*-null space property, another sufficient condition to recover 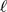-sparse vectors was proposed in [12]. However, there always exist some signals that do not satisfy these conditions but still can be recovered. Specifically, in real-world applications, the known support often contains some errors. The existing sufficient conditions can not reflect accurately the recoverability of modified-CS in many cases. Therefore, it is necessary to develop alternative techniques for analyzing the recoverability of modified-CS.

In this paper, a sufficient and necessary condition (SNC) on the recoverability of modified-CS is derived. Then, we discuss the recoverability of modified-CS in a probabilistic way. The main advantage of our work is that, for a randomly given vector 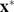 with 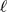 nonzero entries, the exact recovery percentage of modified-CS can be computed explicitly under a given matrix 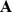 and a randomly given 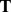 that satisfied 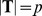 but includes 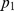 errors, where 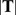 denotes the size of the known support 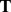. Hence, this paper provides a quantitative index to measure the reliability of modified-CS in real-world applications. Simulation experiments validate our results.

## Materials and Methods

### 1 A Sufficient and Necessary Condition for Exact Recovery

In this subsection, a SNC on the recoverability of modified-CS is derived. Firstly, we give some notations in the follows. The support of vector 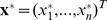 is denoted by 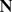, i.e. 
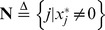
. Suppose 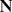 can be split as 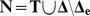, where 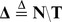 is the unknown part of the support and 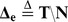 is set of errors in the known part support 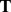. The set operations 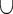 and 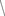 stand for set union and set difference respectively. Let 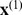 denote the solution of the model in (2) and 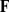 denote the set of all subsets of 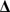. A SNC on the recoverability of modified-CS is given in the following theorem, which is an extension of a result in [16].

**Theorem 1** *For a given vector* 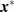 *,* 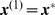 *, if and only if* 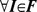 *, the optimal value of the objective function of the following optimization problem is greater than zero, provided that this optimization problem is solvable:*

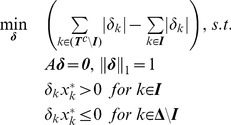
(3)where 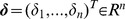.

The proof of this theorem is given in Appendix S1.

**Remark 1:** For a given measurement matrix 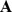, the recoverability of the sparse vector 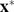 based on the model in (2) depends only on the index set of nonzeros of 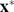 in 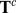 and the signs of these nonzeros. In other words, the recoverability relies only on the sign pattern of 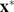 in 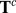 instead of the magnitudes of these nonzeros.

**Remark 2:** It follows from the proof of Theorem 1 that, even if 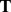 contains several errors, Theorem 1 still holds.

**Remark 3:** Recently, the recoverability analysis of the modified-CS were reported in [6] and [12]. However, we establish a sufficient and necessary condition for the modified-CS to exactly reconstructs a sparse vector, which differs from the sufficient conditions proposed in these works.

### 2 Probability Estimation on Recoverability of Modified-CS

In this subsection, we utilize Theorem 1 to estimate the probability that the vector 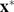 can be recovered by modified-CS, i.e., the conditional probability 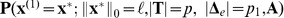, where 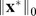 is defined as the number of nonzero entries of 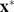, 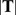 and 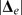 denote the size of 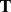 and 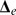 respectively. This probability reflects the recoverability of modified-CS, and is hereafter named as recovery probability.

Let 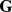 denote the index set 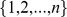, it is easy to know that there are 
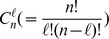
 index subsets of 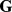 with size 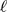. We denote these subsets as 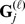, 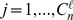. For each 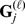, there are 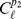 subsets with size 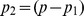. We denote these subsets as 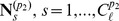. At the same time, for the set 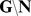 (the index set of the zero entries of 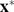), there are 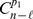 subsets with size 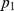. These subsets are denoted as 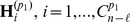. Firstly, we discuss the estimation of the recovery probability under the following assumption.

**Assumption 1** *The index set* 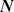 *of the* 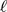 *nonzero entries of* 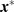 *can be one of the* 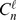 *index sets* 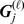 *,* 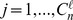 *, with equal probability. The index set* 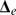 *of* 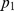 *errors in known support can be one of the* 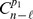 *index sets* 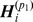 *,* 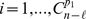 *, with equal probability. The index set* 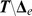 *of* 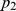 *nonzero entries can be one of the* 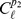 *index sets* 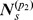 *,* 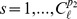 *, with equal probability. All the nonzero entries of the vector* 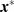 *take either positive or negative sign with equal probability.*

For a given vector 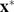 and the known support 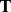, there is a sign column vector 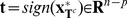 in 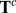. The recoverability of the vector 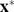 only relates with the sign column vector 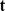 (see Remark 1). Under the conditions that the index set of the nonzero entries of 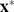 is 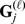 and the known support 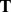 is 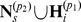, it is easy to derive that 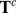 contains 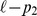 indexes of the nonzeros of 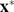, where 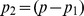. Then there are 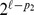 sign column vectors. Among these sign column vectors, suppose that 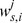 sign column vectors can be recovered, then 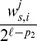 is the probability of a vector 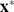 being recovered by solving the modified-CS. Hence, following Assumption 1, the recovery probability is calculated by
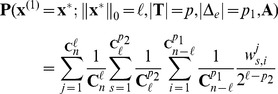
(4)where 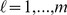, 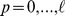 and 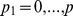. Because the measurement matrix 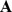 is known, we can determine 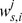 in (4) by checking whether the SNC (3) is satisfied for all the 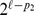 sign column vectors corresponding to the index set 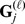, 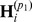 and 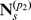.

Because many practical situations such as Electroencephalogram (EEG) signals in wavelet domain do not completely satisfy those assumptions in “Assumption 1”. we further extend our analysis to more general case. Without loss of generality, we have the following assumption

**Assumption 2** *The index set* 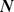 *of the* 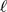 *nonzero entries of* 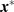 *can be one of the* 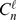 *index sets* 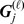 *,* 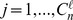 *, with probability* 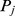 *. The index set* 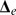 *of* 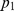 *errors in known support can be one of the* 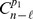 *index sets* 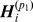 *,* 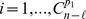 *, with probability* 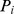 *. The index set* 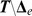 *of* 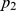 *nonzero entries can be one of the* 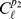 *index sets* 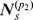 *,* 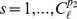 *, with probability* 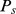 *. All the nonzero entries of the vector* 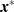 *take either positive or negative sign with probability* 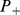 *or* 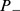 *respectively.*

Similarly, suppose the index set of the nonzero entries of 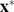 is 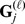 and the known support is 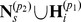, there are 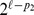 sign column vectors. Since all the nonzero entries of the vector 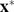 take either positive or negative sign with probability 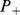 or 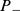 respectively, the probability of the sign pattern of vector 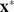 equals one of 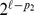 sign column vectors is 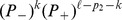, where 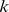 denotes the number of negative signs in this sign column vector and 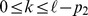. Obviously, there are 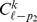 sign column vectors that has 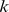 negative signs. Among these vectors, suppose that 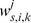 sign column vectors can be recovered, then 
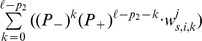
 is the probability of the vector 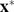 being recovered by solving the modified-CS. Hence, under the Assumption 2, the recovery probability is calculated by
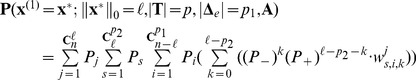
(5)where 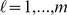, 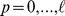 and 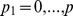. Because the measurement matrix 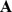 is known, we can determine 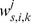 in (5), 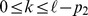, by checking whether the SNC (3) is satisfied for all the 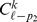 sign column vectors corresponding to the index set 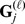, 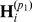 and 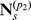.

**Remark 4:** Equation (4) is a special case of equation (5) under the equal probability assumption.

However, the computational burden to calculate (5) increases exponentially as the problem dimensions increase. For each sign column vector 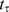 and the corresponding index set 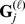, 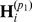 and 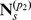, we denote the quads 
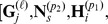
 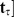, where 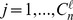, 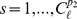, 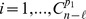 and 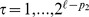. Suppose 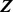 is a set composed by all the quads, there are 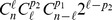 elements in set 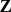. For each element of set 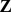, if the sign column vector 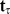 can be recovered by modified-CS with a given measurement matrix 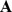 and know support 
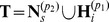
, we call the quad can be recovered. In (5), the estimation of recover probability need to check the total number of quads in set 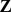. When 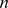 increases, the computational burden will increase exponentially. To avoid the computational burden problem, we state the following Theorem.

**Theorem 2** *Suppose that* 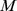 *quads are randomly taken from set* 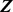 *, where* 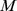 *is a large positive integer (* 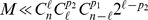 *), and* 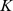 *of the* 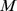 *quads can be recovered by solving modified-CS. Then*

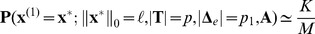
(6)The proof of this theorem is given in Appendix S2.

**Remark 5:** In real-world applications, by sampling randomly 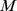 sign vectors with 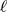 nonzero entries, we can check the number of the vectors that can be exact recovered by modified-CS with a random known support 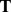 whose size is 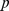 but contains 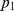 errors. Suppose 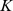 sign vectors can be recovered, the recovery probability 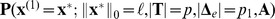 can be computed approximately through calculating the ratio of 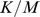.

**Remark 6:** It is well-known that certifying the restricted isometry property is hard, while based on the proposed method, the recoverability probability that reflects the recoverability of modified-CS can be computed explicitly.

From the proof of Theorem 2, the sampling numbers 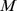, which controls the precision in the approximation of (6), is related to the two-point distribution of 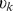 other than the size of 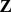. Thus, there is no need for 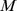 increasing exponentially as 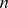 increases.

## Results and Discussion

In this section, simulation examples on both synthesis data and real-world data have been conducted to demonstrate the validity of our theoretical results.

***Example 1*** *:* In this example, the conclusion in Theorem 2 are demonstrated.

**Figure 1:**
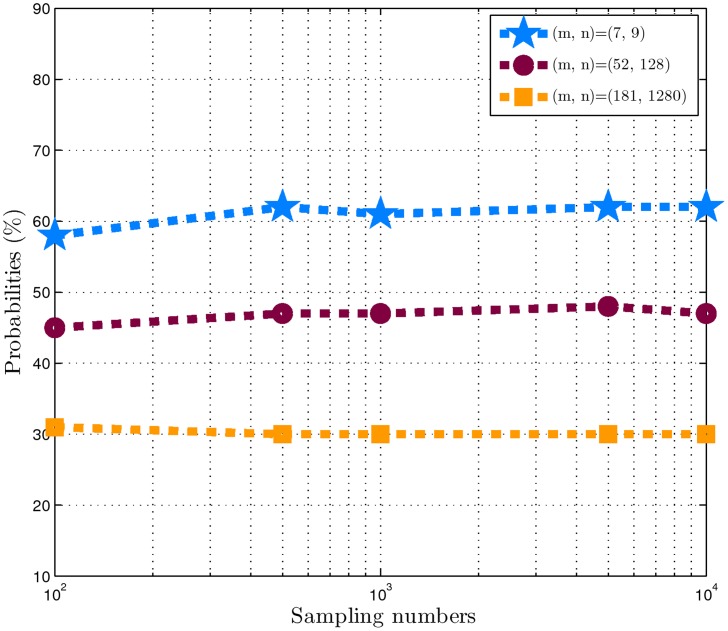
Probabilities curves obtained in example 1. The horizontal axis represents the sampling numbers. The vertical axis represents the probabilities 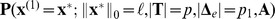 obtained by (6).The three curves from the top to the bottom correspond to 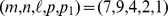, 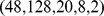 and 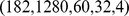 respectively.

According to the uniform distribution in [−0.5, 0.5], we randomly generate three matrices 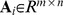 (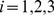) with (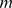, 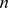) = (7, 9), (48, 128) and (182, 1280) respectively. For matrices 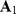, 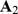 and 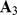, we set (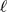, 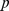, 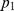) = (4, 2, 1), (20, 8, 2) and (60, 32, 4) respectively. As 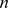 increases in their three cases, the number of sign vectors increases exponentially. For example, for 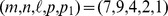, 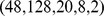, the set 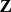 contains approximately 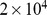 and 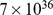 elements respectively. Hence, for their three cases, we estimate the probabilities 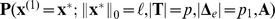 by the sampling method. For each case, we sample 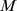 = 100, 500, 1000, 5000, 10000 respectively. The resultant probability estimates depicted in Fig. 1 indicate that 1) the estimation precision of the sampling method is stable in our experiments with different samplings. Therefore, we only need a very few samplings to obtain the satisfied estimation precision in real-world applications; 2) as 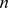 increases in three cases, the sampling 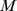 don't increase exponentially.

***Example 2*** *:* Suppose 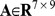 was taken according to the uniform distribution in [−0.5, 0.5]. This example contains two parts in which the recovery probability estimates (4) and (5) are considered in simulation, respectively.

**Figure 2:**
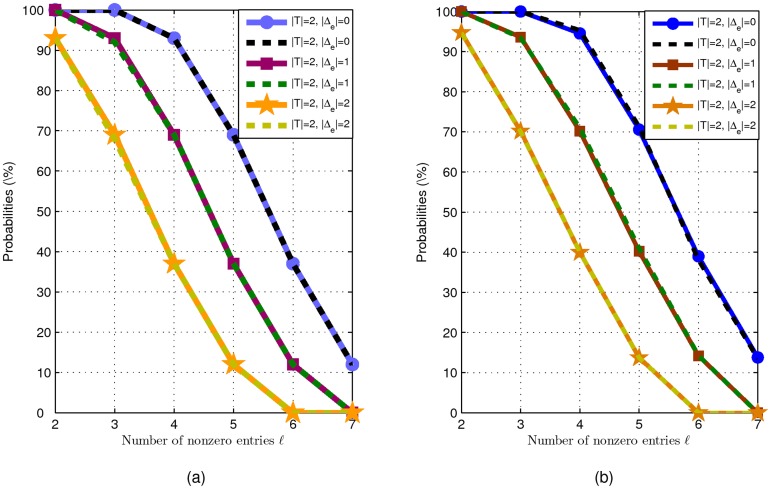
Comparison of theoretical results (solid curves) and simulation results (dotted curves) on recovery probability. Figure (a) shows the experimental results in part I) and figure (b) shows the ones in part II). In both figures, the three pairs of solid and dotted curves from the top to the bottom correspond to 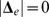, 1, 2 respectively.

1. All nonzero entries of the sparse vector 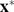 were drawn from a uniform distribution valued in the range [−1, +1]. Without loss of generality, we set 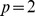. For a vector 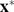 with 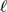 nonzero entries, where 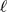 = 2, 3,…, 7, we calculated the recovery probabilities by (4), where 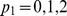 respectively. For every 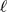 (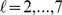) nonzero entries, we also sampled 1000 vectors with random indices. For each vector, we solved the modified-CS with a randomly given 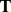, whose size equals to 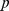 but contains 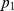 errors, and checked whether the solution is equal to the true vector. Suppose that 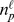 vectors can be recovered, we calculated the ratio 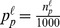 as the recovery probability 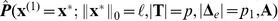. The experimental results are presented in Fig. 2(a). Therein, solid curves denote the theoretic recovery probability estimated by (4). Dotted curves denote probabilities 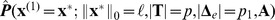. Experimental results show that the theoretical estimates fit the simulated values very well.
2. Now we consider the probability estimate (5). We suppose that all nonzero entries of the sparse vector 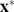 were drawn from a uniform distribution valued in the range [−0.5, +1]. Obviously, the nonzero entries of the vector 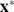 take the positive sign with probability 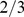 or the negative sign with probability 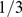. Similarly, we set 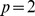. For a vector 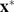 with 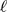 nonzero entries, where 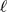 = 2, 3,…, 7, we randomly generate the probabilities 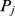 where 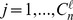. For an index set 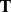 whose size equals to 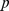 but contains 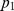 errors, we randomly generate the probabilities 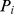 where 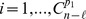 and 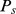 where 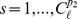. The recovery probabilities are calculated by (5), where 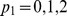 respectively. For every 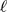 (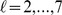) nonzero entries, we also sampled 1000 vectors 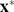 and 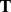 that satisfy the assumption 2 with the above-generated probabilities. For each vector and 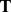, we solved the modified-CS and checked whether the solution is equal to the true vector. Finally, the 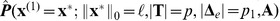 can be calculated with the same way in the part I. We present the experimental results in Fig. 2(b). Therein, solid curves denote the theoretic recovery probability estimated by (5). Dotted curves denote probabilities 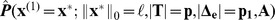. Experimental results show that in the general case, the theoretical estimates also fit the simulated values very well.

***Example 3*** *:* In this example, we test on real-world ECG reconstruction to demonstrate the accuracy of the probability estimation by (5).

Firstly, eight ECG data have been chosen from the MIT-BIH arrhythmia database [17] as the test signals. Each data file includes two-channel ambulatory ECG recordings, and each channel contains 650000 binary data instances in a 16-bits data format, including the index and amplitude. In our simulation, ECG vector 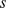 is extracted from the original data at the window size 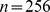. A random sparse binary matrix [18] is used as our sensing matrix 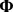 and we use D6 Daubechies wavelet dictionary 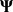 to represent ECG segment, i.e.,
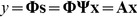
(7)It is well-known that vector 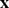 is not strictly sparse, but can be approximated by 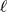-sparse vector. Therefore, to obtain the 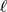-sparse approximation 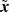 of vector 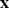, we calculate the standard derivation 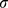 of the high-frequency coefficients in vector 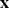 and shrink the coefficients whose magnitudes are less than 3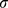 to zero. We define the theoretic recovery of ECG segment 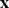 as the SNC (3) can be satisfied for the sign pattern *sign*(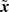). On the other hand, for the recovery of a compressible vector 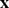, the best one can expect is that the solution of modified-CS and 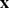 have their nonzero components at the same locations [19]. Considering the noise contamination, we think the ECG segment 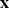 is recovered in practice if the solution of modified-CS and 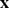 have the overwhelming majority (e.g. 95%) of their nonzero components at the same locations.

**Figure 3:**
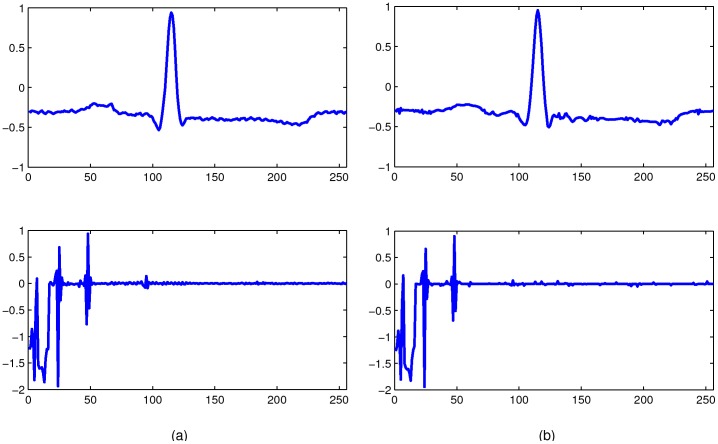
An original segment of record No.100 in the MIT-BIH arrhythmia database and the reconstructed one in time and wavelet domain. Figures (a) and (b) show the original and the reconstructed one respectively.

**Figure 4:**
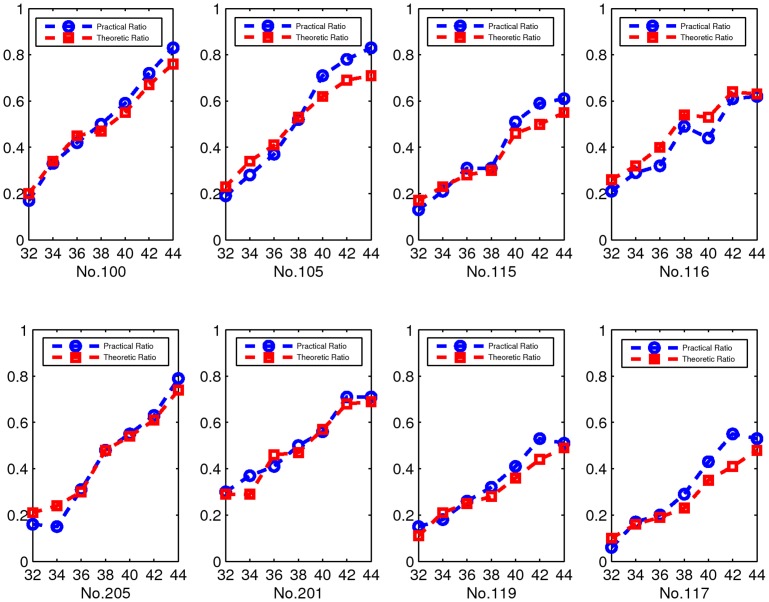
Probabilities curves obtained in example 3. The horizontal axis of each subfig represents the sampling numbers. The vertical axis represents the recovery probabilities. Red curves are the theoretic probability curves; Blue curves are the practical probability curves.

Hence, we randomly extracted 100 segments from each ECG data. According to the Theorem 2, we can estimate the recovery probability of modified-CS through calculating how many segments can be recovered, i.e., the recovery ratio. In our experiment, we suppose 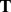 is the index set of low-frequency coefficients in vector 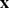. On the one hand, we check the SNC (3) for all the sign patterns *sign*(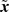) of these segments to obtain the theoretic recovery ratio; on the other hand, we obtain the practical recovery ratio by checking whether these ECG segments can be recovered in practice. For illustration, an original segment of record No. 100 in the MIT-BIH arrhythmia database and its wavelet coefficients are plotted in Fig. 3(a). At the same time, the reconstructed ones in time and wavelet domain are shown in Fig. 3(b). We present the experimental results of eight ECG data in Fig. 4. Therein, red curves denote the theoretic recovery probabilities. Blue curves denote practical recovery probabilities. Experimental results show that the proposed probability estimation is very accurate.

## Conclusion

In this letter we study the recoverability of the modified-CS in a stochastic framework. A sufficient and necessary condition on the recoverability is presented. Based on this condition, the recovery probability of the modified-CS can be estimated explicitly. It is worth mentioning that Theorem 1 can be easy to extend to weighted-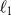 minimization approach that was proposed in [20] for nonuniform sparse model. Moreover, the recovery probability estimation provides alternative way to find (numerically) the optimal set of weights in weighted-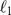 minimization approach, which has the largest recovery probability to recover the signals.

## Supporting Information

Appendix S1 **Proof of Theorem 1.**
(PDF)

Appendix S2 **Proof of Theorem 2.**
(PDF)
