# Supplementary material for: Recoverability Analysis for Modified Compressive Sensing with Partially Known Support
Source: PLoS One. 2014 Feb 10;9(2):e87985. doi: 10.1371/journal.pone.0087985 (PMC3919832; doi:10.1371/journal.pone.0087985)
Supplement: Appendix S1 — Proof of Theorem 1. (PDF) [file pone.0087985.s001.pdf]

APPENDIX S1
PROOF OF THEOREM 1

Proof: **Necessity:** Suppose that $\mathbf{x}^{(1)} = \mathbf{x}^*$. Thus $\mathbf{x}^*$ is the optimal solution and $\|\mathbf{x}^*_{\mathbf{T}^c}\|_1$ is the optimal value of the optimization problem in (2).

For a subset $\mathbf{I} \in \mathbf{F}$, when (3) is solvable, there is at least a feasible solution. For a feasible solution $\boldsymbol{\delta}$ of (3), it can be proved that $\mathbf{x}^* + t\boldsymbol{\delta}$ is a solution of the constraint equation of (2), where t is a constant. In the following, we suppose $t < 0$ with sufficiently small absolute value. Then we have

$$\begin{aligned}\|\mathbf{x}^*_{\mathbf{T}^c} + t\boldsymbol{\delta}_{\mathbf{T}^c}\|_1 &= \sum_{k\in\mathbf{I}} |x^*_k + t\delta_k| + \sum_{k\in\boldsymbol{\Delta}\backslash\mathbf{I}} |x^*_k + t\delta_k| + \sum_{k\in T^c\backslash\boldsymbol{\Delta}} |t\delta_k| \\ &= \sum_{k\in\mathbf{I}} |x^*_k| - |t|\sum_{k\in\mathbf{I}} |\delta_k| + \sum_{k\in\boldsymbol{\Delta}\backslash\mathbf{I}} |x^*_k| + |t| \sum_{k\in\boldsymbol{\Delta}\backslash\mathbf{I}} |\delta_k| + |t| \sum_{k\in T^c\backslash\boldsymbol{\Delta}} |\delta_k| \\ &= \|\mathbf{x}^*_{\mathbf{T}^c}\|_1 + |t|\,(\sum_{k\in(\mathbf{T}^c\backslash\mathbf{I})} |\delta_k| - \sum_{k\in\mathbf{I}} |\delta_k|)\end{aligned} \tag{8}$$

Since $\mathbf{x}^*$ is the optimal solution of the optimization problem (2), it follows from (8) that

$$\|\mathbf{x}^*_{\mathbf{T}^c}\|_1 + |t|\,(\sum_{k\in(\mathbf{T}^c\backslash\mathbf{I})} |\delta_k| - \sum_{k\in\mathbf{I}} |\delta_k|) > \|\mathbf{x}^*_{\mathbf{T}^c}\|_1 \tag{9}$$

Thus,

$$\sum_{k\in(\mathbf{T}^c\backslash\mathbf{I})} |\delta_k| - \sum_{k\in\mathbf{I}} |\delta_k| > 0 \tag{10}$$

The necessity is proved.

Sufficiency: Suppose that $\mathbf{x}^\dagger$ is a solution of the constraint equation in (2), which is different from $\mathbf{x}^*$. Then $\mathbf{x}^\dagger$ can be rewritten as

$$\mathbf{x}^\dagger = \mathbf{x}^* + t^*\boldsymbol{\delta}, \tag{11}$$

where $\boldsymbol{\delta} = \frac{(\mathbf{x}^*-\mathbf{x}^\dagger)}{\left\|\mathbf{x}^*-\mathbf{x}^\dagger\right\|_1}$, $t^* = -\left\|\mathbf{x}^* - \mathbf{x}^\dagger\right\|_1 \neq 0$. Therefore $\boldsymbol{\delta}$ satisfies $\|\boldsymbol{\delta}\|_1 = 1$.

Now we define an index set $\mathbf{I}$,

$$\mathbf{I} = \{k | k \in \boldsymbol{\Delta},\ sign(x^*_k) = sign(\delta_k)\}. \tag{12}$$

From (11), we have

$$\begin{aligned}
\left\|\mathbf{x}^{\dagger}_{\mathbf{T}^c}\right\|_1 &= \|\mathbf{x}^*_{\mathbf{T}^c} + t^*\boldsymbol{\delta}_{\mathbf{T}^c}\|_1 \\
&= \sum_{k\in\mathbf{I}} |x^*_k + t^*\delta_k| + \sum_{k\in\boldsymbol{\Delta}\setminus\mathbf{I}} |x^*_k + t^*\delta_k| + \sum_{k\in\mathbf{T}^c\setminus\boldsymbol{\Delta}} |t^*\delta_k| \\
&\geq \sum_{k\in\mathbf{I}} |x^*_k| - |t^*|\sum_{k\in\mathbf{I}} |\delta_k| + \sum_{k\in\boldsymbol{\Delta}\setminus\mathbf{I}} |x^*_k| + |t^*| \sum_{k\in\boldsymbol{\Delta}\setminus\mathbf{I}} |\delta_k| + |t^*| \sum_{k\in\mathbf{T}^c\setminus\boldsymbol{\Delta}} |\delta_k| \\
&= \|\mathbf{x}^*_{\mathbf{T}^c}\|_1 + |t^*| \, (\sum_{k\in(\mathbf{T}^c\setminus\mathbf{I})} |\delta_k| - \sum_{k\in\mathbf{I}} |\delta_k|)
\end{aligned} \tag{13}$$

It can be easily proved that for the defined index set $\mathbf{I}$ in (12), $\mathbf{I} \in \mathbf{F}$ and $\boldsymbol{\delta}$ is a feasible solution of (3). From the condition of the theorem, we have

$$\sum_{k\in(\mathbf{T}^c\setminus\mathbf{I})} |\delta_k| - \sum_{k\in\mathbf{I}} |\delta_k| > 0. \tag{14}$$

Combining (13) and (14), we have that for any solution $\mathbf{x}^{\dagger}$ of the constraint equation in (2), which is different from $\mathbf{x}^*$,

$$\left\|\mathbf{x}^{\dagger}_{\mathbf{T}^c}\right\|_1 > \|\mathbf{x}^*_{\mathbf{T}^c}\|_1. \tag{15}$$

Hence, $\mathbf{x}^*$ is the unique optimal solution of (2). Thus, $\mathbf{x}^{(1)} = \mathbf{x}^*$. The sufficiency is proved.
