# Supplementary material for: Recoverability Analysis for Modified Compressive Sensing with Partially Known Support
Source: PLoS One. 2014 Feb 10;9(2):e87985. doi: 10.1371/journal.pone.0087985 (PMC3919832; doi:10.1371/journal.pone.0087985)
Supplement: Appendix S2 — Proof of Theorem 2. (PDF) [file pone.0087985.s002.pdf]

APPENDIX S2
PROOF OF THEOREM 2

Proof: Suppose $\mathbf{Z}$ can be split as $\mathbf{Z} = \mathbf{Z}_e \cup \mathbf{Z}_f$, where $\mathbf{Z}_e$ denotes the set composed by the S_w quads that can be recovered, $\mathbf{Z}_f = \mathbf{Z} \setminus \mathbf{Z}_e$. For a quad ζ, we have

$$\mathbf{P}(\zeta \in \mathbf{Z}_e) = \frac{S_w}{C_n^\ell C_\ell^{p_2} C_{n-\ell}^{p_1} 2^{\ell - p_2}} \tag{16}$$

Now we define a sequence of random variables υ_k using the set of of quads $\mathbf{Z}_e$

$$\upsilon_k = \begin{cases} 1, \ \zeta_k \in \mathbf{Z}_e \\ 0, \ \zeta_k \in \mathbf{Z}_f \end{cases} \tag{17}$$

where $k = 1, 2, ..., \zeta_k$ is a quad randomly taken from $\mathbf{Z}$.

From (16), it follows that $\mathbf{P}(\upsilon_k = 1) = S_w / C_n^\ell C_\ell^{p_2} C_{n-\ell}^{p_1} 2^{\ell - p_2}$, $\mathbf{P}(\upsilon_k = 0) = 1 - \mathbf{P}(\upsilon_k = 1)$. Therefore, $\upsilon_k, k = 1, 2, ...$ are independent and identically distributed random variables with the expected value $E(\upsilon_k) = S_w / C_n^\ell C_\ell^{p_2} C_{n-\ell}^{p_1} 2^{\ell - p_2}$.

According to the law of large numbers (Bernoulli) in probability theory, the sample average $(1/M) \sum_{i=1}^{M} V_i = K/M$ converges towards the expected value $E(\upsilon_k)$, where V_i is a sample of the random variable υ_k. It follows that when M is sufficiently large

$$E(\upsilon_k) = S_w / C_n^\ell C_\ell^{p_2} C_{n-\ell}^{p_1} 2^{\ell - p_2} \backsimeq \frac{K}{M} \tag{18}$$

The theorem is proven.
